# Commonalities in the Association between PPARG and Vitamin D Related with Obesity and Carcinogenesis

**DOI:** 10.1155/2016/2308249

**Published:** 2016-08-08

**Authors:** Borja Bandera Merchan, Francisco José Tinahones, Manuel Macías-González

**Affiliations:** ^1^Unidad de Gestión Clínica Endocrinología y Nutrición, Instituto de Investigación Biomédica de Málaga (IBIMA), Complejo Hospitalario de Málaga (Virgen de la Victoria), Universidad de Málaga, 29010 Malaga, Spain; ^2^CIBER Pathophysiology of Obesity and Nutrition (CB06/03), 28029 Madrid, Spain

## Abstract

The PPAR nuclear receptor family has acquired great relevance in the last decade, which is formed by three different isoforms (PPAR*α*, PPAR*β*/*δ*, and PPAR *ϒ*). Those nuclear receptors are members of the steroid receptor superfamily which take part in essential metabolic and life-sustaining actions. Specifically, PPARG has been implicated in the regulation of processes concerning metabolism, inflammation, atherosclerosis, cell differentiation, and proliferation. Thus, a considerable amount of literature has emerged in the last ten years linking PPARG signalling with metabolic conditions such as obesity and diabetes, cardiovascular disease, and, more recently, cancer. This review paper, at crossroads of basic sciences, preclinical, and clinical data, intends to analyse the last research concerning PPARG signalling in obesity and cancer. Afterwards, possible links between four interrelated actors will be established: PPARG, the vitamin D/VDR system, obesity, and cancer, opening up the door to further investigation and new hypothesis in this fascinating area of research.

## 1. Introduction

There are three subtypes of PPARG, known as PPARG1, PPARG2, and PPARG3. It has been established that PPARG2 leads in potency as a transcription factor [1]. PPARG performs its functions mainly through PPARG1 and PPARG2 [2]. Moreover, it shares lots of additional features with its other counterparts. Concerning that, the parallelism found between the PPARG system and the vitamin D/vitamin D receptor (VD/VDR) system will be further explored later on.

In order to modulate gene expression, the PPAR NRs family, and specifically the PPARG, after binding with either natural or synthetic ligands, heterodimerizes with the Retinoid X Receptor (RXR) as vitamin D receptor (VDR) does.

Later on, the complex PPARG-RXR translocates to the nucleus in order to get attached to PPREs (PPAR Response Elements), genome nucleotides sequences wherefrom the PPARs will coordinate the expression or repression of some genes involved in metabolism, immunity, differentiation, or cellular proliferation, to cite some [3–6].

Once in the nucleus, several molecules known as corepressors and coactivators, which show histone modifying activities by themselves [7], bind the PPARG-RXR complex, showing some control over the genetic expression-repression interplay. Some known corepressors are SMRT or NCOR. When it comes to coactivators, we can mention p300/CRRB-binding protein (CBP) or SRC/p160 [8]. Importantly, differential recruitment of coactivators implies different gene expression patterns [9], wherefrom it can be deduced that the corepressors and coactivators comprise another gene expression regulatory point which is worth studying. PPREs are normally found in the promoter of those genes, which is regulated by PPARG activity [3]. The direct nucleotide sequences which PPARG-RXR will be bound to are known as DR-1 motifs (direct hexanucleotide repeats) of PPRE [8]. Some PPARG target genes are those codifying CD36, FABP4 (Fatty Acid Binding Protein 4), adiponectin, or the CCAAT/enhancer binding protein *α* [10], all being genes involved in adipose tissue homeostasis. However, afar of its adipose functions PPARG is also vital for development of some important organs such as heart and the placenta [11].

## 2. The PPARG Physiology

PPARG behaves as a transcription factor, as many other nuclear receptors (NRs) do. Then, it modulates the expression and repression of a myriad of genes involved in metabolic homeostasis, regulating energy expenditure and storage [12, 13]. Some PPARG target genes are those codifying CD36, FABP4 (Fatty Acid Binding Protein 4), adiponectin, or the CCAAT/enhancer binding protein *α* [10], all being genes involved in adipose tissue homeostasis. However, afar of its adipose functions PPARG is also vital for development of some important organs such as heart and the placenta [11]. Although most research on PPARG has been focused on its metabolic action, some of them are neurogenesis, osteogenesis, cancer, or cardiovascular disease [14]. Such pleiotropism of actions gives us a clue of the relevance of this transcription factor regarding health and disease. We know for instance that universal PPARG deletion and life are not compatible [11].

The considerable host of actions performed by PPARG can be compared to those of vitamin D and VDR [15], which has been implicated in neurologic disorders [16–18], autoimmune pathologies [19–21], cardiovascular disease [22], diabetes mellitus [23, 24], psoriasis [15] or infectious disease [25, 26], and, above all of what is mentioned, cancer [27, 28].

## 3. PPARG and Obesity

Much has been already written about PPARG signalling and its role in conditions such as obesity or diabetes. In obesity, PPARG orchestrates adipocyte maturation and differentiation, harmonising the role of many other players in that process [29]. Remarkably, it is the only known factor, which is completely necessary and sufficient for the adipocyte differentiation process to occur [11, 30]. This nuclear receptor acts, then, as a master regulator of adipogenesis.

In addition, it is widely known that PPARG has an important whole-body insulin-sensitizer role. For example, muscle-PPARG knocked-out mice are insulin resistant [31]. In adipose tissue, PPARG deletion leads to increases in bone mass, lipoatrophy, and insulin resistance (IR) [32]. In the same fashion, PPARG induces the proliferation of adipocytes progenitors into mature adipocytes and diminishes the osteoblasts population likewise [33].

The specific deletion of PPARG in liver conduces to IR and decrease of hepatic fat depots [34]. Even in macrophages, the presence of PPARG is important to keep adequate insulin sensitivity levels throughout the body [35, 36]. It is then easy to deduce that one of the main objectives of PPARG activity is the insulin sensitivity maintenance through different tissues.

Thiazolidinediones (TZD), a family of synthetic PPARG agonist widely used in diabetes treatment, show clear improvements in insulin sensitivity, enhanced adipocyte differentiation, reduction of leptin levels, and upregulation of adiponectin [37].

Contrary to the catabolic actions elicited by the PPAR*α* and PPAR*δ*, the PPARG is in charge of anabolic functions. As we have already addressed, adipogenesis and lipid storage are some of them. Illustrating this, a high-fat feeding augments PPARG expression while fasting diminishes it [38].

Remarkably, PPARG performs different functions in metabolically sick rodents and metabolically healthy ones. In disease, PPARG activation seems to improve metabolic parameters, but in the healthy population its downregulation shows antiobesity effects [39].

In the same way, more different effects have been described in metabolic health and disease regarding PPARG expression. For instance, in healthy subjects a high-fat meal greatly induced the expression of PPARG while the same high-fat feeding diminished PPARG expression in a group of morbidly obese patients [40].

In like manner, an indirect correlation between IR and PPARG expression, measured by glucose status, HOMA-IR index, and insulin levels, can be set in morbidly obese persons, whose visceral adipose and muscle tissues show less PPARG expression as IR increases [40].

During placentation and intrauterine development, the PPARG gene methylation patterns could be altered by maternal nutrition, which actually exerts long-term effects upon the receptor status in the offspring, as indicated very recently by Lendvai et al. [41]. This is preliminary evidence about the early programming of our lifelong metabolism set points through nutritional inputs, which could easily leave us susceptible to obesity and metabolic disease in later stages of life.

## 4. PPARG and Cancer

**Figure 1:**
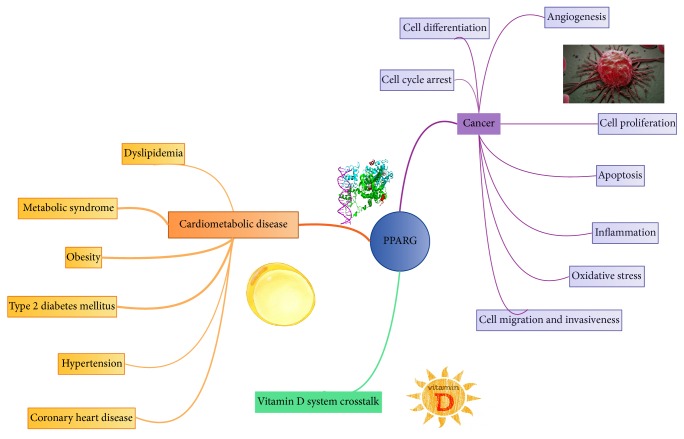
PPARG actions: PPARG plays an important role in cardiometabolic disease and cancer. The noteworthy crosstalk between vitamin D system and PPARG is also considered. Arrow's width exemplifies the level of consistency found in the literature regarding each association in the mind picture.

PPARG is highly expressed in lung, prostate, colorectal, bladder, and breast tumours [42]. Furthermore, we can find in the literature compelling evidence for PPARG having antineoplastic actions in colon, prostate, breast, and lung cancers [43, 44], which happen to be the most prevalent forms of cancer in occident (Figure 1).

Solid evidence backs up that epigenetic events frequently found in cancer can hamper nuclear receptors responsiveness toward their ligands. In that respect, increased levels of corepressor NCOR in prostate cancer can silence the expression of target genes and constitute a potential epigenetic lesion, which selectively distorts the actions of PPARG/PPAR*α* [45].

In the same line, PPARG promoter methylation in colorectal carcinoma (CRC) is associated with poor prognosis [46]. This transcriptional silencing of PPARG is operated through HDAC1 (Histone Deacetylase 1), EZH2 (Enhancer of Zeste 2 Polycomb Repressive Complex 2 Subunit), and MeCP2 (Methyl CpG Binding Protein 2) recruitment, leading to repressive chromatin states that eventually increase cell proliferation and invasive potential [46]. Correspondingly, APC^min/+^ mice which have undergone PPARG genetic ablation demonstrate increased colon tumour growth [47].

In the literature, some mutations and variations in PPARG expression have been associated with cancer in our specie [48, 49]. Beyond that, its expression comprises an independent prognostic factor in CRC [50, 51].

Apart from epigenetics, we should not lose sight of the fact that metabolic syndrome, insulin resistance, obesity, and inflammation, importantly interrelated conditions in which PPARG has modifying and regulatory actions, increase cancer risk [52–59], which adds weight to PPARG and cancer research (Figure 1).

There is some evidence linking PPARG agonist's actions to better cancer treatment responsiveness as well. PPARG agonist Rosiglitazone, in this phase II clinical trial, raised the radioiodine uptake in differentiated thyroid cancer [60].

IFN-*β* treated pancreatic cancer cells were more affected when Troglitazone was added to the therapy, showing synergistic effects between IFN-*β* and TGZ [61]. But it is necessary to be careful in some studies, in which PPARG agonist like Rosiglitazone acts as a great promoter of hydroxybutyl nitrosamine-induced urinary bladder cancers [62].

In the following paragraphs, we will review what we know about the specific molecular actions of PPARG in cancer biology. Cell cycle arrest, cell differentiation, angiogenesis, proliferation, invasiveness, migration capacity, apoptosis, inflammation, and oxidative stress should be evaluated.

### 4.1. Cell Cycle Arrests

Some evidence suggests that PPARG and its agonists have the ability to interfere with the cellular cycle and then, likely, with malignancies development.

In renal cell carcinoma, Troglitazone (TGZ) was able to induce G2/M cell cycle arrest via activation of p38 MAPK (Mitogen-Activated Protein Kinase) [63].

In human pancreatic cancer cells the same phenomenon is observed: PPARG is able to trigger cell cycle arrest of the malignant cells through activation by thiazolidinediones [64].

Through PPARG activation, its ligands increase the expression of the cyclin-dependent kinase inhibitors p21 [64, 65] and p27 [65–69], enhance the turnover of *β*-catenin, and downregulate the expression of cyclin D1 [70–74].

### 4.2. Differentiation

In vitro activation of PPARG by its ligands correlates with increased expression of carcinoembryonic antigen (CEA), E-cadherin, developmentally regulated GTP-binding protein 1 (DRG), alkaline phosphatase, or keratins, all of them being molecules expressed in well differentiated cells, opposing to the undifferentiated cell state commonly found in most cancers [48, 64, 75–77].

Tontonoz et al. gave us the first evidence about the effectiveness of PPARG ligands inducing differentiation in human cancer cells, concretely in liposarcoma cancer cells [75]. Again, in human liposarcoma, treatment with Troglitazone raised the level of differentiation of its cells [78].

More evidence that PPARG enhances terminal differentiation in cells is reviewed in papers of Grommes et al. and Koeffler, respectively [43, 44].

### 4.3. Angiogenesis

It is common knowledge that angiogenesis is a vital step in malignant development. The complex process by which new vessels are formed, angiogenesis, has been feverishly studied as a new possible target in cancer treatment.

In vitro and in vivo angiogenesis-modulating functions have been described for PPARG [79]. In spite of that, differential effects regarding angiogenesis have been observed for PPARG in vitro and in vivo, showing either pro- or antiangiogenic actions dependent on cell context [80–85]. PPARG agonist can also enhance VEGF expression in cancer cells, as some studies reveal [86, 87].

The mechanisms deciding whether PPARG will act as a proangiogenic factor or as an antiangiogenic one are still elusive to us, but we believe that cellular context and environment are likely the controllers of such process.

### 4.4. Proliferation

Antiproliferative actions are also attributed to PPARG and its ligands. TZD, for example, has shown antiproliferative effects [88, 89].

Modulation of PPARG can have differential effects on carcinogenesis depending on the cellular microenvironment [90]. Therefore, depending on the cellular environment PPARG can behave as a proliferative or antiproliferative factor, as happened with angiogenesis.

Tumour cells are frequently in shortage of polyunsaturated fatty acids. Docosahexaenoic acid (DHA), a well-known ligand of the PPAR family, has been shown to reduce tumour proliferation in lung tumour cell cultures [91]. Along with that, DHA in breast cancer cells diminishes proliferation and increases apoptosis [92, 93].

In prostate cancer, PPARG ligand activation effect was assessed in a phase II clinical trial. The results showed a hampered cancer cell growth [94].

Eukaryotic initiation factor 2 is a target of inhibition for PPARG agonists (i.e., thiazolidinediones). Such factor inhibition, which is mediated in a PPARG-independent way, truncates the translation process [95].

In liposarcoma patients, treatment with Rosiglitazone increased the necessary time to double tumour volume in this clinical trial [96]. In other studies, however, Troglitazone (another member of the thiazolidinedione family) had low or no effects in prostate cancer [97] or breast or colorectal cancer [98, 99].

### 4.5. Apoptosis

The combined effect of an RXR agonist and Troglitazone curtailed gastric cancer cells proliferation in vitro by enhancing apoptotic mechanisms [100].

PPARG agonists increased the expression of PTEN [101–105], BAX, BAD [106, 107], and the turnover of the FLICE inhibitory protein (FLIP) [108, 109], known for its antiapoptotic role.

Conversely, PPARG agonists can inhibit BCL-X_L_ and BCL-2 expression [107, 110], PI3K activity, and AKT phosphorylation [101, 111, 112] and restrain the activation of JUN N-terminal protein kinase [107]. It is worth mentioning that many of those actions were elicited in a PPARG-independent manner. The exact mechanisms by which these effects are performed are still unknown.

### 4.6. Inflammation

Nowadays, it is common knowledge in the scientific community that chronic inflammation promotes cancer. The milieu found in chronic inflammation acts as a facilitator for carcinogenesis and cancer development [52, 113]. This has been shown in colorectal, liver, bladder, lung, and gastric neoplasms [114, 115] and investigated in several more. The range of processes in which inflammation partakes in carcinogenesis goes from cell growth and survival, metastasis and cell invasion, treatment response, angiogenesis, and tumour immunity [115, 116].

There is evidence of PPARG having anti-inflammatory activity in several cell lines [117, 118]. In models of experimentally induced colitis PPARG expressed in macrophages is capable of inhibiting inflammation [119].

It is widely known that some PPAR ligands such as omega-3 fatty acids EPA and DHA have anti-inflammatory properties. Those and other natural and synthetic ligands could be used in the future as chemopreventive agents in a vast range of conditions linked to inflammation, that is, cancer [105, 120, 121].

Activation of PPARG by its ligands reduces cytokines such as TNF*α* and NF-*κβ* in monocytes, turning down the inflammatory milieu [120, 122].

The epigenetic process of sumoylation has been linked to PPARG transrepression of inflammation. After ligand activation, PPARG binds to a SUMO protein (Small Ubiquitin-like Modifier) and both join a nuclear corepressor complex, reducing the proinflammatory gene expression [123].

The NF-*κβ* transcription factor has repeatedly been associated with tumour development and thriving [52]. Interacting with this factor, PPARG inhibits the genesis of proinflammatory molecules such as IL-6, TNF, and MCP1 through transrepression [3, 117].

Again, a word of caution must be said due to the seemingly tumour-promoting effects of PPARG found sporadically [124–127]. Therefore, it seems as if the effects carried on by the cell depend of cell context and environment. Environment is, usually, at the helm of cellular functions.

### 4.7. Oxidative Stress

PPARG has demonstrated an antioxidant effect [128, 129]. SOD (Superoxide Dismutase) expression might well be regulated by PPAR because a PPRE is found in the Cu/Zn-SOD promoter [40].

IR found in diabetes mellitus and metabolic disease is certainly correlated with increased oxidative stress, which eventually could lead to an increased risk of cancer through nongenomic carcinogenesis [130–133].

In macrophages, PPARG mediates some notable abilities: uptake and reverse transport of cholesterol, macrophage subtype specification (enhancing the M2 macrophage phenotype, which is associated with higher insulin sensitivity and lower inflammation levels), and anti-inflammation properties [36, 134, 135].

Postprandial hypertriglyceridemia is associated with lower PPARG expression in metabolic syndrome patients while in healthy subjects the same “insult” leads to overexpression of PPARG [136]. We could hypothesize that since the PPARG system is injured in the metabolically ill patients, after an oxidative stress insult (a high-fat feeding), it cannot respond, leaving us more susceptible to oxidative actions and its consequences (hypothesis coined as “*nuclear receptor exhaustion theory*”). In the healthy group, the PPARG would perfectly be capable of managing the lipid storage and would act as an oxidative stress buffer.

### 4.8. Cell Migration and Invasiveness

Less evidence is available with respect to invasiveness and PPARG. However, we should pay attention to some preliminary data.

The PPARG gene modulates the invasion of cytotrophoblast into uterine tissue, which could be a novel indicator of some invasion-related function of PPARG [137].

Going further, this study by Yoshizumi et al. showed how PPARG ligand thiazolidinedione (TZD) is able to inhibit growth and metastasis of HT-29 human colon cancer cells, via the induction of cell differentiation. The use of the TZD drives to G1 arrest, in association with a great increase in p21Waf-1, Drg-1, and E-cadherin expression [77].

Paradoxically, molecules with PPARG antagonist actions are able to inhibit invasiveness and proliferation of some cancer cell lines [26, 138–140]. Again, one nuclear receptor can exert one or just the opposite function depending on the cellular environment and ligand exposure.

## 5. Connecting the Dots: PPARG, Vitamin D System, Obesity, and Cancer

Often in biology and medicine research, we tend to focus on the individualities of separated molecules or molecule systems in order to explain their functions, forgetting the intermolecular communication, which is ever-present in every biological system. More frequent than not, that separateness gives us a rather limited perspective of the matter at hand. For instance, the interconnectedness of biology systems and the emerging properties of such interconnectedness should be further examined and taken into account.

The crosstalk between different NRs, the “dance” and messages they give one another, is recently becoming an exciting new area which will be explored. This is the case of the VDR/VD and the PPARG system, in which both have been shown to be involved in some relationship we do not utterly understand yet.

### 5.1. PPARG and VDR/VD System: Commonalities in Cancer

Noteworthy, great parallelism exists between PPARG and the VDR/VD system regarding its protective role in carcinogenesis. There are a vast number of studies describing the anticancer properties of vitamin D. The majority of them are brilliantly analysed in this review by Feldman et al. [28].

Vitamin D has been extensively associated with anti-inflammatory actions [141–143], apoptotic mechanisms [144–150], antiproliferative functions [151–159], prodifferentiation effects [160–166], antiangiogenic properties [167–171], a potential role-managing invasion and metastasis [172–184], microRNA modulation [185–189], and even some role in the Hedgehog signalling pathway modulation [190]. Remarkably, most of those actions have been attributed to PPARG signalling in a somewhat lesser extent, as reviewed in this work. Such similarity and overlap in anticancer actions are worth studying.

Moreover, there is enough evidence to assert that epigenetic events can influence both PPARG and VDR/VD systems behaviour.

In this study, Fujiki et al. showed that in a diabetic mouse model PPARG promoter methylation levels are higher than those of the control mice [191], along with the possibility of methylation reversal when the animals were exposed to 5AZA (5′-aza-cytidine). At least three messages can be drawn from this study: (1) the PPARG system is susceptible to epigenetic regulation, (2) diabetes and other metabolic conditions could alter the PPARG epigenetic landscape and then disrupt its proper functioning, and (3) this disruption can be reversed by drug-induced changes or, likely, by lifestyle changes.

The vitamin D system is likewise susceptible to epigenetic regulation [192–195] and, interestingly, in cancer this epigenetic repression of the vitamin D system is almost always present [196–204], which compellingly leaves the door opened to the possibility of the same phenomena happening in the PPARG system.

In fact, PPARG promoter hypermethylation is a prognostic factor of adverse outcome in colorectal cancer [46, 205]. Higher levels of PPARG promoter methylation were found in advanced tumour stages while earlier stages showed lower methylation levels. This suggests that as happens with vitamin D, advanced cancer stages can epigenetically repress PPARG expression and then nullify its antineoplastic actions.

### 5.2. The PPARG/VDR Crosstalk: What an Interesting Conversation!

Some studies have clearly shown the existence of some communication between PPARG and VD/VDR. Interestingly, potent VDRE (Vitamin D Response Elements) have been discovered in human PPAR*δ* promoter, which opens the door to VDR/VD influence over the PPAR system [206]. In the opposite direction, some studies have demonstrated the ability of PPARG to bind VDR and inhibit vitamin D-mediated transactivation [207]. This data might be an indicator of bidirectional or reciprocal actions of both systems influencing each other, which have deep implications and introduce new and interesting questions to ponder upon.

Even between PPAR subtypes some modulation of expression have been found: PPAR*δ* could repress PPAR*α* and PPARG gene expression [208], illustrating the complexity of PPAR system regulation.

In the adipocyte cell, the VD/VDR system has shown anti-PPARG activity, inhibiting its expression and then adipogenesis [209, 210], which is contradictory with the commonly found proadipogenesis effects of vitamin D [211], at least in human. The factors leading to either pro- or antiadipogenesis effects are completely uncharted.

In melanoma cell lines, administration of calcitriol and several PPAR ligands modified the expression of both PPARG and VDR, demonstrating again this intriguing connection [212]. Sertznig et al. conclude in this article that calcitriol and some PPAR ligands can inhibit proliferation of the human melanoma cell line MeWo [213].

### 5.3. PPARG and VD/VDR System: Metabolic Commonalities

We are about to discuss the metabolic effects of vitamin D and their analogy with those of PPARG, establishing again the parallelism.

As contradictory as it seems, VDR or CYP27B1 knocked-out mice show great fat mass loss [211] while obesity in humans is commonly associated with poor vitamin D plasmatic levels [214]. Actually, an indirect relationship between Body Mass Index (BMI) and 25OHD3 has been amply described in the literature [215].

In addition, low plasmatic vitamin D levels are associated with increased risk of type 2 diabetes mellitus (T2DM) independently of BMI [24] and with hypertension, dyslipidemia (DLP), and metabolic syndrome (MS) [216, 217]. Besides, vitamin D deficiency predisposes to diabetes in animal models, while its supplementation prevents the disease [214]. Concerning PPARG, we have extensively discussed before in the review its orchestrating actions regarding adipogenesis and adipocyte metabolism. Both calcitriol (the active form of vitamin D) and PPARG seem to oppose metabolic homeostasis disruption.

Another paradoxical event is found in the fact that in humans calcitriol enhances adipogenesis while in mice the same hormone diminishes it via downregulation of C/EBP*β* mRNA and upregulation of CBFA2T1 (a corepressor) [218, 219]. With reference to PPARG, it enhances adipogenesis [10].

In human subcutaneous preadipocytes, calcitriol elicits actions impressively similar to those of PPARG in adipocyte maturation and differentiation. For instance, calcitriol is able to increase the expression of the enzyme Fatty Acid Synthase (FASN) increasing lipogenesis in like manner as PPARG [210].

The* storage capacity theory* introduces the idea that lipid storage capacity and the ability of PPARG to manage the processes leading to lipid storage are limited. As to that, when the organism reaches a lipid level threshold lipotoxicity shows up, PPARG is no more capable of lipid handling, and the harmful hormonal environment of obesity starts to spread through the organism [220].

Transferring the same concept of “*nuclear receptor exhaustion*” to VD/VDR anticancer actions we could establish a parallelism. It has shown that the VD/VDR is epigenetically downregulated in late cancer stages but overexpressed or normally expressed in early stages [221, 222]. As the aforementioned studies show, in those later stages epigenetic downregulation of the VD system molecules occurs, leaving it unable to exert its antineoplastic functions properly. Is obesity, as cancer does with vitamin D, acting as a negative epigenetic driver when it comes to PPARG signalling? That could answer why in most morbidly obese patients expression of PPARG is greatly lower in comparison to healthy subjects.

Accordingly, PPARG1 and PPARG2 expression in visceral adipose tissue (VAT) from morbidly obese (MO) subjects is significantly downregulated when compared to metabolically healthy subjects [223]. Not only that, in insulin resistant MO subjects PPARG expression is even lower [220] compared with noninsulin resistant MO patients, whichever interestingly correlates with the lower vitamin D levels found in MO with IR compared to their insulin sensitive counterparts [24]. Somehow, the metabolic impairment caused by insulin resistance is able to deteriorate both PPARG and VD/VDR system. The underlying mechanism behind this deterioration should be further studied.

A disrupted VDR/VD system leads mice to loss of fat deposits and great increase of energy expenditure. In relation to that, VDR^−/−^ mice increase the expression of UCP1 (uncoupling protein 1 or Thermogenin) twenty-five-fold [211], with the consequent energy consumption. Is vitamin D, along with PPARG, an energy-conserving and metabolic homeostasis-maintaining hormone?

However, adipose tissue is not the only one affected by disruption of the VD system. A shortage of calcitriol in rats was related with increased skeletal muscle ubiquitination and loss of total muscle mass [224]. On the PPARG side, its activation through TZD in growing pigs increased muscle fiber oxidative capacity independently of fiber type [225]. Overexpression of PPAR*δ* in mice almost doubles the animal endurance and exercise capacity [226]. We should not lose sight of the important role the muscle has in obesity and metabolic disease pathogenesis, being a potential target for calcitriol and PPAR modulating actions.

Taken all data together, the vitamin D system seems to team up with PPARG in order to maintain proper metabolic homeostasis. Notwithstanding, in some occasions this love relationship breaks apart and both partners seem to bother one another in ways that we utterly ignore but, likely, have something to do with epigenetic regulation.

## 6. Conclusions

The PPARG transcription factor has been classically associated with metabolic homeostasis and lipid storage functions. Recently, newfound anticancer actions are assigned to this nuclear receptor.

However, its anticancer actions are not always consistent; in some studies some oncogenic effects have been described. We believe that cellular environment is the guiding factor behind PPARG actions and cells are controlled “from outside in.” In alignment with this, the PPARG and other nuclear receptors would only be “cellular effectors,” carriers of outside messages of health or disease.

When a “disease threshold” is reached, in either obesity or cancer, PPARG and VDR expression, respectively, diminishes. However, in early stages of those diseases, the expression of those nuclear receptors is higher than normal. Derived from these observations, we have coined the so-called “*nuclear receptor exhaustion theory,*” by which, in an early disease stage, nuclear receptors PPARG and VDR counterbalance the harmful effects that obesity and cancer exert upon the organism, their expression being high. However, sadly, if disease progresses, it generates epigenetic silencing mechanisms upon both transcription factors, whose expression decreases radically. This silencing leaves us increasingly susceptible to disease. The positive side is that through drugs or, better yet, lifestyle changes reversal of epigenetic changes is possible.

There is an exciting function overlap between PPARG and VDR/VD system, both of which wield oncoprotective and metabolic actions. Actually, parallel metabolic and anticancer actions are described in the literature, suggesting that they team up to keep at bay those diseases. Maybe the detailed study of this overlap could give us clues in respect to the molecular pathogenesis of important conditions as metabolic disease and cancer. Further study in this new area is necessary to elucidate those questions.

Obesity, a first-order problem in our society, is linked with increased risk of cancer incidence and progression. The debatable factors behind this risk are an increment in oxidative stress, chronic inflammation, poorer vitamin D status, hormone misbalance, and, arguably, PPARG silencing through unknown mechanisms. As we know, PPARG and the vitamin D system play conjunctly a yet-to-elucidate role in cancer, so it is not surprising at all that their hypothetical epigenetic repression in obesity could be another mechanism linking this metabolic disorder to malignancies.

It has been shown, both in vitro and in vitro, that the tumours are capable of epigenetically silencing both the vitamin D and the PPARG system. This silencing could lead to the deterioration of their anticancer and metabolic actions.

**Figure 2:**
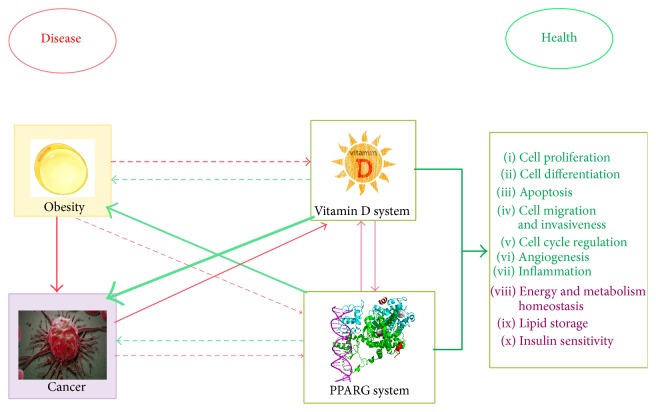
The four players: this figure shows the interrelation between the four players: obesity, cancer, vitamin D system, and PPARG. Red arrow: harmful effects, which contribute to disease. Green arrow:positive effects, which contribute to health. Disease perpetuates itself damaging both nuclear receptors. Arrow's width is in proportion with the strength and consistency of each association found in the literature. Dashed line: yet-to-be determined, preliminary, or hypothetical effects. Continuous line: in vitro/in vivo demonstrated effects. Right box: in green, actions mainly attributed to vitamin D, and in purple, actions classically attributed to PPARG. However, it is known that both agents exert every action illustrated in this box, in a higher or lower extent.

Finally, a worse known crosstalk between the two NRs exists. Its usefulness, purpose, and message are (almost) utterly unexplored to us and should be studied more diligently. The interrelation, reciprocity, and interdependence of all four actors examined here might be the starting point of new fascinating research linking epigenetic signalling and two of the most hurtful diseases of our time (Figure 2).

## Additional Points

Design is literature review across preclinical studies, descriptive studies, analytic studies, and reference lists of selected studies. The author focused mainly on systematic and narrative reviews. Data sources are medline (Pubmed), Jábega 2.0 (Málaga University Search Engine Software), Gerión search engine, and screening of citations and references. Regarding eligibility criteria, we focused on papers published in magazines considered to be in the first impact factor quartile without restrictions regarding publishing date. Keywords are PPARG; Obesity; Transcription factor; Vitamin D; Calcitriol; Vitamin D Receptor; Epigenetics; Nuclear Receptor; Cancer; Methylation.
